# Study on the Impact of LDA Preprocessing on Pig Face Identification with SVM

**DOI:** 10.3390/ani15020231

**Published:** 2025-01-16

**Authors:** Hongwen Yan, Yulong Wu, Yifan Bo, Yukuan Han, Gaifeng Ren

**Affiliations:** College of Information Science and Engineering, Shanxi Agricultural University, Jinzhong 030801, China; 17652525251@163.com (Y.W.); 13453109054@163.com (Y.B.); 18536494757@163.com (Y.H.); 15343511954@163.com (G.R.)

**Keywords:** SVM, LDA, individual identification, intelligent management of pig breeding

## Abstract

In this study, the implementation of traditional machine learning models in the intelligent management of swine is explored, focusing on the impact of LDA preprocessing on pig facial recognition using an SVM. Through experimental analysis, the kernel functions for two testing protocols, one utilizing an SVM exclusively and the other employing a combination of LDA and an SVM, were identified as polynomial and RBF, both with coefficients of 0.03. Individual identification tests conducted on 10 pigs demonstrated that the enhanced protocol improved identification accuracy from 83.66% to 86.30%. Additionally, the training and testing durations were reduced to 0.7% and 0.3% of the original times, respectively. These findings suggest that LDA preprocessing significantly enhances the efficiency of individual pig identification using an SVM, providing empirical evidence for the deployment of SVM classifiers in mobile and embedded systems.

## Simple Summary

Porcine facial recognition technology is critical for the intelligent husbandry and precision management of pigs. There is a significant demand for the deployment of this technology in mobile and embedded applications within small to medium-sized pig farms. Consequently, to enhance the model’s applicability to these farms, this study integrates LDA preprocessing into the conventional approach. Experimental results indicate that the model is well suited for small and medium-sized pig farms, facilitating the intelligent transformation of swine management practices.

## 1. Introduction

Pork is one of the primary sources of meat globally, playing a significant role in the food industry while also serving as a cornerstone of agricultural economies in many countries. The pig farming industry not only generates substantial employment opportunities but also drives the development of related sectors, such as feed, veterinary medicine, and equipment manufacturing. With the rapid advancement of intensive farming worldwide, the importance of implementing intelligent breeding and precise management in pig farms has become increasingly evident. Among these, individual identification and behavior analysis of pigs have emerged as critical components of intelligent management. Several research teams have carried out research in the fields of individual identification, automatic detection, activity and behavior detection, diet and drinking water monitoring, and abnormal behavior recognition of pigs. These research areas demonstrate a growing interest in the development of technologies for monitoring and analyzing various aspects of pig behavior and welfare. In the midst of this, the identification of individuals and the recognition of behaviors are essential components for a wide range of studies. Currently, research methods for pig identification and behavior recognition fall into three categories. Initially, RFID technology was utilized to enhance reader efficiency and establish a traceability system for the pig industry, significantly benefiting automatic feeding, pork quality management, slaughter supervision, and production safety [1,2,3,4,5,6]. The second category involves traditional machine learning models. Researchers have explored the use of models such as k-means [7,8], LDA [9], KNN [10,11], random forest [12,13,14], and SVMs [15,16,17] to improve intelligent pig management. Significant progress has been made in individual identification, huddling behavior, walking speed detection, exercise quantification, and sleep statistics.

The third paradigm pertains to computer vision technology that predominantly emphasizes the application and enhancement of deep learning models. In the field of industrial inspection, Chen [18] and Xu [19], respectively, utilized partial convolution networks and generative adversarial networks to detect specular highlights in industrial metal images, effectively reducing inaccuracies caused by surface highlight reflections. He [20] proposed a VGSG method to align text and visual modalities, enabling the retrieval of target pedestrian images based on textual descriptions, with significantly improved efficiency and effectiveness in cross-modal alignment compared to existing methods. Pan [21] designed a miner fatigue recognition system that employs ResNeXt-50 and GRU for feature extraction and Transformer+ for multi-modal data fusion, achieving an accuracy of 93.15%.Researchers have employed architectures such as the BPNN [22], Faster R-CNN [23], GoogleNet [24], the YOLO series [25,26,27,28,29], and transformers to investigate individual identification, alimentary and hydration monitoring, locomotion activities like climbing and crossing, as well as aggressive behaviors. This paradigm necessitates substantial computational power, hence its current predominant use in laboratory settings. For the intelligentized management of small and middle-sized pig farms, there is a significant demand for mobile terminals and embedded applications. Facilitating the intelligent advancement of these farms requires the provision of suitable technology and equipment. While the practical utilization of the first paradigm is restricted, the third paradigm’s extensive computational and financial requirements hinder its widespread adoption in small and medium-sized pig farms. Consequently, the second paradigm, which offers improved identification accuracy and operational efficiency at a lower cost and is supported by extensive research foundations, provides a suitable option for the intelligentized development of small and middle-sized pig farms.

Hence, this research investigated the impact of LDA preprocessing on pig face recognition utilizing SVM. The combination of LDA and SVM preprocessing achieved an accuracy rate of 86.3% in the LDA + SVM testing program, which is 2.64 percentage points higher than the standalone SVM method. The training duration for the LDA + SVM approach was 98 ms, substantially lower than the 12,823 ms required for the single SVM method, representing merely 0.7% of its time expenditure. The recognition time for the LDA + SVM method was 1 ms, compared to 329 ms for the SVM method, equating to just 0.3% of the latter’s duration, thus significantly enhancing operational efficiency. The experimental results indicate that employing the LDA + SVM approach for pig face recognition not only enhances recognition accuracy but also markedly improves operational efficiency, thereby providing theoretical underpinnings for the development of real-time and portable embedded pig face recognition systems.

Maselyne et al. [30] introduced a methodology for delineating feeding visits via RFID registration for growing pigs at feeding stations. Their experimental outcomes demonstrated that using two tags per pig yielded an average sensitivity of 83%, specificity of 98%, precision of 97%, and accuracy of 75%, thereby enabling the automated and precise logging of feeding data for growing pigs. Moreover, Maselyne et al. [31] developed a high-frequency radio frequency identification system designated for monitoring individual pigs’ drinking behaviors. The system exhibited a sensitivity of 92%, specificity of 93%, precision of 90%, and accuracy of 93%, thereby enhancing the productivity and economics of the swine industry and promoting pig health and welfare. Additionally, Maselyne et al. engineered an HFRFID system validated for tracking individual pigs’ feeding behaviors at round troughs within a group housing environment, showing promise for monitoring growing–finishing pigs’ feeding patterns in commercial settings [32]. Kapun et al. [33] implemented the ultra-high frequency radio-frequency identification system to track the daily movements of pigs, achieving approximately 80% sensitivity at feeding troughs and playback devices, as well as about 60% at the drinkers. Their findings highlighted the system’s capability to record visit times with a higher data density compared to video or direct observation methods. Chen T. Y. et al. [34] utilized a radio-frequency identification technique to gather dietary information in pigsty feeding zones, revealing a sensitivity of 71.1%, specificity of 87.1%, and accuracy of 88.8%, thus enabling remote pig farming monitoring and significantly reducing farmers’ labor costs. Zhu Weixing et al. [35] developed a pig behavior monitoring system that is embedded with RFID technology and uses the Linux system based on ARM processors, which facilitated continuous monitoring and data collection in the pig breeding area, demonstrating excellent real-time performance and stability. Zhu Jun et al. [36] constructed a digital pig breeding platform incorporating RFID, intelligent control, and network transmission technologies, achieving a seamless integration of automated precision feeding, environmental control, production management, and visual monitoring. Other researchers tested various scenarios using predictive models like LASSO regression and random forest to forecast the weight of pigs aged between 159 and 166 days [37], while others used random forest and generalized linear regression to test different scenarios in predicting piglets’ physiological temperature, but the prediction errors are relatively high [38]. Furthermore, auto-regression and enhanced local linear embedding models were applied in real farm environments to forecast pig weight, while Fast-rcnn [39], SSD [40], and environmental models [41] were applied to study pigs’ posture changes and subsequently analyze their behaviors. Yan Hongwen [42] combined feature pyramid attention with the architecture of tiny-YOLO to enhance the detection of multiple pigs in different environmental conditions. Subsequently, Yan Hongwen [43] utilized YOLOV3 as the base model and integrated spatial and channel attention mechanisms to create sub-models of specific attention for detecting various behaviors of pigs living in groups. Hu Zhiwei [44] created a dual-attention mechanism that integrates both channel and spatial attention by utilizing Residual Neural Network-50 and Residual Neural Network-101 as the backbone networks, which was then applied within the feature pyramid network framework to achieve instance-level detection of live pigs under different conditions.

With the advent of GPU technology, the precision capabilities of computer vision techniques have become increasingly evident. The demand for mobile and embedded applications in managing small to medium-sized farms has surged. However, deep learning models impose significant hardware demands, complicating their widespread adoption. Conversely, while RFID technology offers straightforward simulations in management scenarios [45,46], it necessitates physical tags such as ear cuts and ear tags, causing discomfort to pigs. Moreover, the installation of these tags is labor-intensive for workers and contravenes animal welfare principles, and low-frequency RFID systems are ineffective in handling signals from multiple pigs simultaneously, with high-frequency RFID systems suffering from limited identification ranges [47]. Consequently, RFID technology is being phased out. Traditional machine learning models, despite their compatibility with mobile and embedded application standards, still have room for improvement concerning identification accuracy and processing speed.

The current feature selection and extraction techniques are mainly divided into two categories: feature selection methods and feature extraction methods. Common feature selection methods, such as variance thresholding [48] and recursive feature elimination (RFE) [49], remove redundant or irrelevant features by filtering existing features without generating new ones. Although these methods can improve model performance, reduce computational complexity, and mitigate the risk of overfitting, they have limitations in handling feature correlation, information loss, and applicability, which may affect the final detection accuracy. Therefore, they are not suitable for complex tasks such as facial feature extraction in pig identification.

Feature extraction methods, such as linear discriminant analysis (LDA), principal component analysis (PCA), independent component analysis (ICA), t-SNE, and multidimensional scaling (MDS), map high-dimensional data into lower-dimensional space while maintaining information integrity and preserving key features of the original data. These methods not only reduce computational load, eliminate noise, and improve recognition accuracy but also retain the global structure and important information of the data, thereby enhancing the overall performance of the model.

However, ICA requires complex matrix or tensor operations, has a slow convergence rate, and its convergence is highly dependent on the selection of the learning rate parameter [50]. Similarly, while t-SNE [51] excels in preserving local structures, it is sensitive to parameters and may lead to the loss of global structural information, making it less ideal for applications that require a comprehensive understanding of global data relationships. MDS [52], on the other hand, has dimensionality selection limitations and is typically used for two- or three-dimensional visualization. Although MDS can operate in higher dimensions, its interpretability and practicality significantly decrease in high-dimensional spaces.

Considering the advantages and disadvantages of the above methods, feature extraction methods offer greater applicability and effectiveness in handling complex image tasks, such as facial feature extraction in pig identification. After our research team studied the impact of PCA preprocessing on pig face recognition, we continued to explore the impact of various feature extraction methods on pig face recognition, with LDA preprocessing being the core focus of this study.

The authors examined the impact of PCA preprocessing on RF for pig individual identification in the literature to promote the application of traditional machine learning models in mobile and embedded systems [53]. Preprocessing with PCA can enhance the effectiveness of RF in identifying individual pigs, resulting in a 2.66% increase in accuracy, 2.76% increase in recall, and 2.81% increase in f1 scores. Additionally, testing time was reduced to 75% of the original duration. To further explore the impact of different preprocessing methods on traditional machine learning models in the field of pig individual identification, this study examined the effect of using LDA as a preprocessing step on the efficiency of pig individual identification using support vector machines (SVMs). There is sufficient evidence supporting the usage of this technology in mobile and integrated applications. The terminology utilized in this document is outlined in Table 1.

## 2. Materials and Methods

### 2.1. Data Acquisition

**Figure 1:**
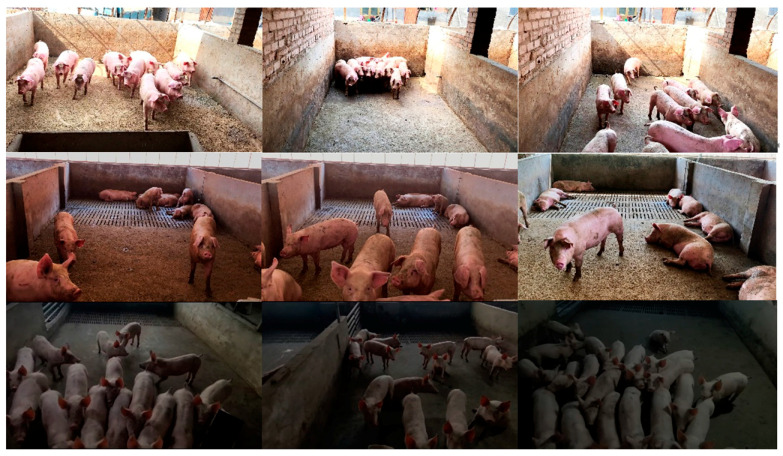
Sample data collection.

Considering the influence of environmental factors such as light intensity and other experimental effects, the data of this study came from two different environments, and most of the data were collected by using fixed and non-fixed camera positions under the side view angle. The images were captured using a Canon EOS M100 camera (Canon Inc., Tokyo, Japan). The first part of pig image data was collected from 9:00 to 14:00 on 1 June 2019 in Dongsong Jiazhuang (111°95′ E, 37°27′ N), Fenyang Market Village, Shanxi Province, China. The ambient temperature was moderate, which was conducive for pig activities, and the conditions were full of sunlight and strong light. This involves capturing images of live pigs in a variety of pen environments in three selected pig farms, each consisting of 10–30 pens with six to eight pigs each, with pen sizes of approximately 3.5 m × 2.5 m × 1 m. A total of 35 video clips were recorded from five pig stalls, with the age of pigs ranging from 20 days to 105 days, and the video clips during the active period of the pigs were mainly recorded during the filming process. The second part of the data was collected at the Laboratory Animal Management Center of Shanxi Agricultural University (112°59′ E, 37°43′ N), Taigu City, Shanxi Province, China, from 10:30 to 12:00 on 13 October 2019. The ambient temperature was moderate, which was convenient for the activities of the pigs, and the shooting was carried out under the conditions of a cloudy day with insufficient light. This phase consisted of capturing video from six pig stalls ranging in age from 20 to 105 days, with a total of 15 pigs involved in all the video segments. In this experiment, 10 pigs in all the videos were selected as experimental objects, and the sample sizes of the training set, the verification set, and the test set were 768, 85, and 250, respectively. A diagram of data acquisition is shown in Figure 1.

### 2.2. Data Redundancy Processing

The image dataset utilized in this experiment comprises image frames extracted from continuous video sequences. The sample images exhibit a high degree of similarity due to data redundancy stemming from the correlation between consecutive frames. Consequently, to mitigate data redundancy, the structural similarity index (SSIM) [54] algorithm is employed to assess the structural similarity, brightness, and contrast between the preceding and subsequent frames. The higher the SSIM value, the higher the similarity between images. After experimental analysis, the images with a similarity threshold greater than 0.8 in the collected live pig images was deleted so as to reduce the occurrence of data redundancy.

Since the structural similarity index measure (SSIM) determines the similarity between two images by measuring the similarity of the two images in three aspects: structure, contrast, and brightness, the algorithm is also composed of a structural comparison function, S(x,y), a contrast comparison function, C(x,y), and a brightness comparison function, L(x,y). The calculation formulas are shown in Formulas (1)–(3):

(1) $$S\left(x,y\right)=\frac{\sigma_{\mathrm{xy}}+C_{3}}{\sigma_{x}\sigma_{y}+{\;C}_{3}}$$

(2) $$C\left(x,y\right)=\frac{2\sigma_{x}\sigma_{y}+{\;C}_{2}}{\sigma_{x}^{2}+\sigma_{y}^{2}+{\;C}_{2}}\;$$

(3) $$L\left(x,y\right)=\frac{2\mu_{x}\mu_{y}+{\;C}_{1}}{\mu_{x}^{2}+\mu_{y}^{2}+{\;C}_{1}}$$

The SSIM index function SSIM(x,y) is obtained by combining the structural comparison function S(x,y), the contrast comparison function C(x,y), and the brightness comparison function L(x,y). The calculation formula is shown in Formula (4).

(4) $$\mathrm{SSIM}\left(x,y\right)=\frac{\left(2\sigma_{x}\sigma_{y}+{\;C}_{1}\right)\left(2\sigma_{\mathrm{xy}}+{\;C}_{1}\right)}{\left(\mu_{x}^{2}+\mu_{y}^{2}+{\;C}_{1}\right)\left(\sigma_{x}^{2}+\sigma_{y}^{2}+{\;C}_{2}\right)}$$

In calculation Formulas (1)–(4), μ_x_ and μ_y_ are, respectively, the average pixel values of image x and image y. σ_x_ and σ_y_ are, respectively, the standard deviations of the pixel values of x and y. σ_xy_ represents the covariance of the pixel values of x and y. C_1_, C_2_, and C_3_ are constants.

### 2.3. Pig Face Recognition Area Standard

**Figure 2:**
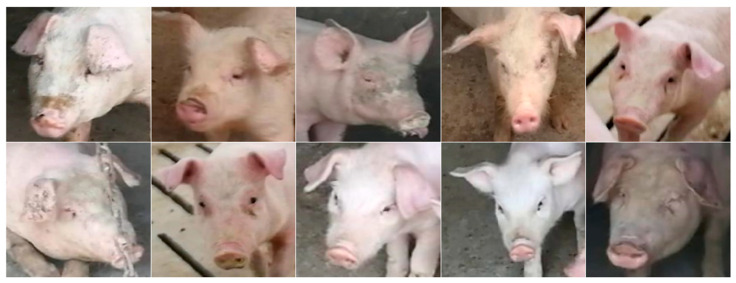
Pig data samples.

When defining the pig face recognition region, Marsot et al. cut pig ears in the collected images, focusing on retaining the features of forehead, eyes, and nose [55]. Similarly, Wang et al. extracted the facial areas of pigs by determining the distance between the ear and chin [56]. Based on these studies and the needs of this experiment, we determined that the image of pig face recognition area was a pig face image with the distance between pig ears as the width and the distance between pig forehead and pig jaw as the height mark, as shown in Figure 2.

### 2.4. Data Processing

In this experiment, the data used are images. Therefore, the input feature vector is composed of the pixel values of the image. The original image size is 1280 (width) × 720 (height), including the three RGB channels of the image. The image needs to go through preprocessing steps before training to remove interference information and increase the complexity of the samples. The main data processing method currently used is normalization and data augmentation. Data augmentation methods can make up for the information loss caused by normalization processing to some extent, effectively improving the robustness and adaptability of the model, so data augmentation is used as the data processing method. The processing process mainly includes size adjustment and data augmentation operations. First, the pig face part of the image is cropped, and the size is adjusted to a fixed 140 (width) × 140 (high) before the data augmentation processing.

**Figure 3:**
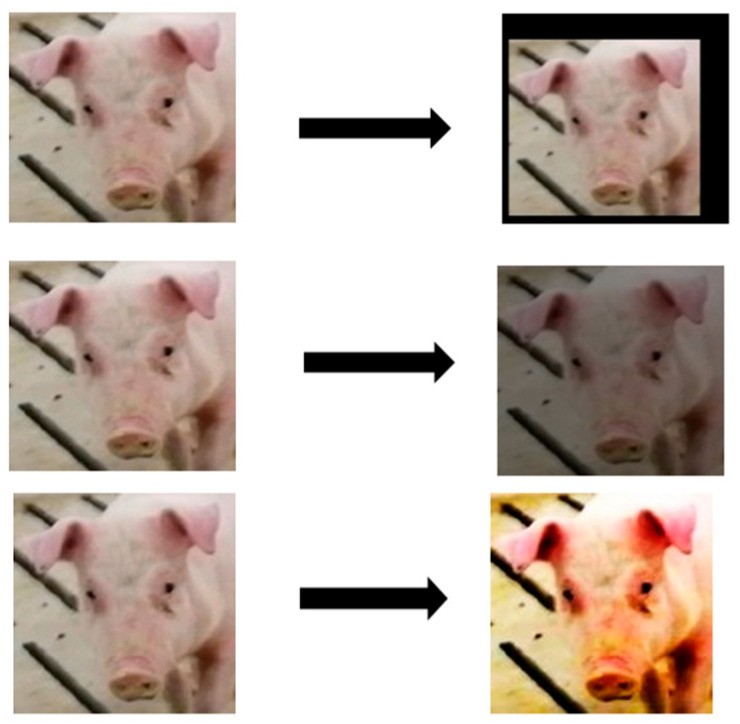
Data augmentation example.

Due to the complexity of pig activity and environmental factors, it is necessary to enhance the data of pig face images after adjusting the size. The robustness and generalization ability of the model can be improved effectively by using data enhancement on the image of the dataset. Considering the sensitivity of LDA dimensionality reduction to image pixel arrangement, it is necessary to use an enhancement method that does not significantly change the position of key features, pixel distribution, and the overall structure of the image. Finally, the random cropping method that can remove background noise while preserving key features is selected, and the brightness adjustment method and the contrast adjustment method that can enhance the adaptability of the model to different illumination brightness and illumination conditions without changing the position of key features and pixel distribution of the image are selected. The data enhancement method we used is shown in Figure 3, which is shown as random cropping, brightness adjustment, and contrast adjustment in turn.

### 2.5. Experimental Running Environment

The experimental setup utilized a computer equipped with a 64-bit Windows operating system, an Intel Core i7-6700 processor, 8 GB of RAM, 6 GB of VRAM, and Python version 3.5 for program development.

Table 2 shows the parameters required for SVM model training. Kernel function represents the type of kernel function used by the model and is set to RBF; C represents the penalty coefficient for misclassified samples and is set to 1.0; degree represents the use times of polynomial kernel function and is set to 3; tol represents the error value of stopping training and is set to 1 × 10^−3^; cache_size represents the cache size used for training and is set to 200 mb, and max_iter represents the maximum number of iterations for model training and is set to 100.

### 2.6. Principle of SVM-Based Pig Face Identification

A support vector machine is a kind of machine learning algorithm, which is mainly based on structural risk minimization, derived from statistical learning theory. It aims to balance model complexity and learning capability using finite sample information, thus achieving optimal generalization. An SVM is resilient to issues like overfitting and local minima during training, making it widely applicable in practical classification and control tasks [57,58,59,60].

An SVM constructs an N-dimensional hyperplane to perform classification, akin to neural networks. Specifically, using an S-shaped kernel function, the SVM model can be equated to a two-layer perceptron neural network. Various kernel functions such as polynomial functions, radial basis functions, and multi-layer perceptron classifiers are employed in an SVM. These kernels transform the optimization of network weights into a quadratic programming problem with linear constraints, ensuring identical parameter values when both functions reach optimal solutions. This method mainly uses Lagrange optimization theory, combined with the application of advanced calculation methods and theoretical frameworks to deal with quadratic convex functions with constraints. Fundamentally, an SVM seeks to identify a predictive variable (attribute) and a transformed attribute (feature), known as feature selection. The SVM model optimally identifies a hyperplane that segregates vector clusters, classifying vectors on different sides into distinct categories, with vectors near the hyperplane serving as support vectors. In 1979, Vapnik proposed an SVM, which describes the hyperplane in a linear form by separating positive and negative samples at the largest interval; he did not focusing on training error but on minimizing generalization error.

The crux of the classification problem lies in delineating positive and negative instances via a conventional classifier. For a training set where data points are vectors of m dimensions, this classifier’s objective is to discern a hyperplane that segregates these vectors. However, data often exhibit linear inseparability, necessitating the use of a support vector machine with a nonlinear kernel function. This approach maps the input space (x⊂R^n^) into a high-dimensional feature space, enabling the construction of an optimal separating hyperplane with robust generalization capabilities. The SVM then selects the hyperplane with the maximum margin within the training set, enhancing generalization for unseen data.

In linear scenarios, the distance between the hyperplane and the nearest positive and negative instances is defined as the margin. The linear SVM output formula is as follows:

(5) u=w×x−b

where w denotes the unit normal vector to the hyperplane, [dimensionless]; p denotes the input feature vector, [dimensionless]; b denotes the classification threshold, [dimensionless].

For the decision boundary, the separating hyperplane is defined by the equation *u* = 0, with the closest points lying on the planes *u* = +1 and *u* = −1. The margin is given by

(6) $$m=\frac{1}{{\left\|w\right\|}^{2}}$$

where w denotes the normal vector to the hyperplane, [dimensionless]; m denotes the margin of the hyperplane, [dimensionless].

Support vector machines (SVMs) can be generalized to handle nonlinear classification problems. The Lagrange multiplier method is employed to compute the output of a nonlinear SVM:

(7) $$u=\sum_{j=1}^{N}y_{j}a_{j}K\left(x_{j},x\right)-b$$

where K denotes the kernel function, which quantifies the similarity or distance between the input vector *x* and all training vectors $x_{j}$, [a]; $y_{j}$ represents the classification tag of the training vector $x_{j}$, [dimensionless]; b represents the threshold for classification, [dimensionless]; j represents the sample size, [dimensionless]; $a_{j}$ represents the Lagrange multiplier for the *j*th sample, [dimensionless]; u represents the discriminant function, [dimensionless].

In this study, SVM was utilized for pig face classification, a multi-classification problem. According to SVM principles, two methodologies exist for multi-class classification:

1. The implementation of the multi-class task utilizes several binary SVM classifiers, and the aggregation of the results of these binary classifiers determines the final output;

2. The SVM refinement process can be modified to compute multi-class decision functions directly. This approach was employed in the study for pig face data classification. Thus, the original problem can be reformulated as follows:

(8) $$min:1/2\overset{k}{\underset{m=1}{\sum }}{\left|\left|w_{m}\right|\right|}^{2}+C\overset{n}{\underset{i=1}{\sum }}\underset{m\neq y_{i}}{\sum }\xi_{i}^{m}$$

where i represents the sample size, [a]; w represents the normal vector to the hyperplane, [dimensionless]; m represents the number of categories, [a]; $y_{i}$ represents the classification tag of the training vector *x_i_*, [dimensionless]; $\xi_{i}$ represents the Lagrange multiplier for the *i*th sample, [dimensionless].

Each base learner classifies the pig samples according to the selected feature sequence, while the method used by the random forest algorithm to make the final classification decision is absolute majority voting. The detailed rules for this voting method are shown below:

(9) $$S.T.\left(w_{i}\cdot x_{i}\right)+b_{i}\geq \left(w_{m}\cdot x_{i}\right)+b_{m}+2-\xi_{i}^{m},\;\xi_{i}^{m}\geq 0$$

where i the represents sample size, [a]; w represents the normal vector to the hyperplane, [dimensionless]; m represents the number of categories, [a]; $x_{i}$ represents the ith sample, [dimensionless]; $\xi_{i}$ represents the Lagrange multiplier for the *i*th sample, [dimensionless].; b represents the threshold for multiple-classification, [dimensionless].

Thus, the decision function $f\left(x\right)=max\left[\left(w_{i}\cdot x_{i}\right)+b_{i}\right]$ can be obtained, and the discriminant result pertains to the *i*th category.

## 3. Comparison Process in the Experiment

### 3.1. Pig Face Identification Test Carried out with an SVM Alone

#### 3.1.1. Determine the SVM Model Parameters

**Figure 4:**
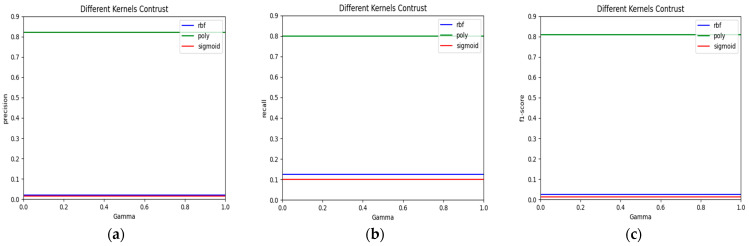
The connection between the evaluation metrics of the SVM model and its kernel function along with the coefficients. (**a**) Accuracy; (**b**) recall rate; (**c**) *f*1 value.

When using an SVM to classify pig face data, the key to establishing the SVM classifier model lies in determining the kernel function and its coefficient. In the research process, the performance of different kernel functions under different coefficients is tested first, and then the optimal kernel functions and their coefficients are selected according to the test results. The functions of the selected support vector machine are RBF radial kernel function, polynomial kernel function, and sigmoid kernel function, respectively, and the kernel function coefficient Gamma is guaranteed to vary between 0.0 and 1.0. The curves illustrating the changes in classification accuracy, recall rate, and *f*1 value are shown in Figure 4.

Figure 4a illustrates how the kernel function coefficient Gamma relates to classification accuracy. The blue line denotes the RBF radial kernel function, while the green line indicates the polynomial kernel function, and the red line represents the sigmoid kernel function. As evident from the figure, the three curves were generally smooth and consistent, indicating that the kernel function coefficient Gamma had a minimal impact on classification accuracy. The polynomial kernel function achieved an accuracy of 82%, whereas the RBF (radial basis function) kernel and the sigmoid kernel functions both had accuracies of approximately 1%.

Figure 4b displays the relationship between the kernel function coefficient Gamma and the classification recall rate. The data indicates that the polynomial kernel function outperformed both the RBF (Radial Basis Function) and sigmoid kernel functions in recall rate for pig face recognition, demonstrating superior identification performance.

Figure 4c depicts the correlation between the kernel function coefficient Gamma and the f1 score. The data show that the polynomial kernel function delivered the best classification results for pig face data. Based on these observations, the polynomial kernel function was selected as the kernel function for the SVM in this experiment.

#### 3.1.2. SVM Model Evaluation Parameters

**Figure 5:**
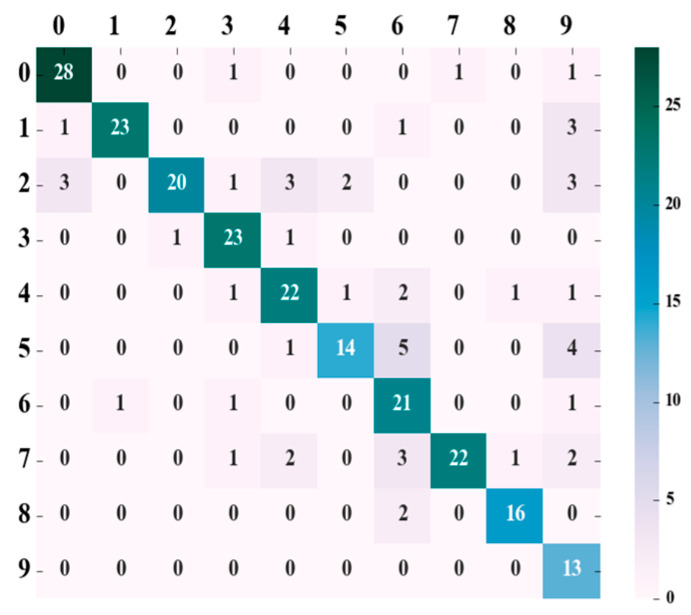
Confusion matrix for SVM prediction results.

The confusion matrix, as an important evaluation tool, provides a detailed representation of a classification model’s predictions across different categories. Analyzing the confusion matrix allows for a more accurate identification of the model’s strengths and weaknesses, particularly when handling imbalanced data, offering a more comprehensive perspective on the model’s performance. To more thoroughly assess the classification model’s performance, this study utilized parameters obtained from early tests and chose the ‘poly’ kernel for the SVM. Before testing on the dataset, the kernel coefficient was set to 0.03. Based on the test results, a confusion matrix was generated, as shown in Figure 5. The process of generating the confusion matrix involves comparing the true labels of the test set with the predicted labels by counting the number of correct and incorrect classifications. This process further allows for the calculation of performance metrics such as accuracy, recall, and f1 score, providing a quantitative evaluation of the model’s classification ability across different categories.

Figure 5 presents the confusion matrix for the SVM model before applying LDA preprocessing. The rows represent the true classes, and the columns represent the predicted classes, with diagonal elements indicating correctly classified instances and off-diagonal elements reflecting misclassifications. The SVM model performs well for classes 0, 1, and 7, with correct classification counts of 28, 23, and 21, respectively. However, some misclassifications are observed, particularly for class 5, where four instances are incorrectly classified as class 4 and class 9, which exhibits a relatively higher misclassification rate. These misclassifications suggest that the model may benefit from additional improvements in differentiating between certain classes, particularly classes 5 and 9.

To further quantify the model’s performance, we calculated the precision, recall, and *f*1-score values for each of the ten pigs, based on the confusion matrix from Figure 5, using Formulas (10)–(12), as presented in Table 3. These metrics provide a deeper insight into the model’s ability to correctly classify instances, as well as its performance in handling misclassifications, particularly in challenging classes like classes 5 and 9.

The precision ratio is defined as

(10) $$precision=\frac{TP}{TP+FP}$$

The recall ratio was defined as

(11) $$recall=\frac{TP}{TP+FN}$$

The recall ratio, also known as the recall rate, exhibited an inverse trend compared to the precision ratio. The *f*1 *score* integrates these two metrics and reflects their respective preferences, with the formula given as follows:

(12) $$f1-score=2\times \frac{precision\times recall}{precision+recall}$$

where *TP* represents the number of positive samples that are actually positive samples, [a]; *FP* represents the number of positive samples that are actually negative samples, [a]; *FN* represents the number of negative samples that are actually positive samples, [a].

Table 3 shows that the SVM model achieved an average classification accuracy of 83.66% for pig face data. The recall rate was 79.53%, and the f1 score was 79.95%, highlighting the model’s effective performance in both identifying and classifying the data.

### 3.2. Experiment of Pig Face Identification with LDA + SVM Pretreatment

#### 3.2.1. Determination of the Target Dimension of LDA

The dimension reduction target of the LDA method was relative to the number of classes in the original data. After going through dimension reduction by LDA, if the number of data class was n and the number of data samples was m, then the number of dimension could only be reduced to n − 1. A matrix to be processed whose dimension is [m, n − 1] was generated after the face images of m pigs were processed by LDA, and the class number of pigs in this study was, respectively, 1~10. In total 10 classes were present, so it was reduced to nine dimensions.

#### 3.2.2. Determination of SVM Parameters in the Optimization Plan

**Figure 6:**
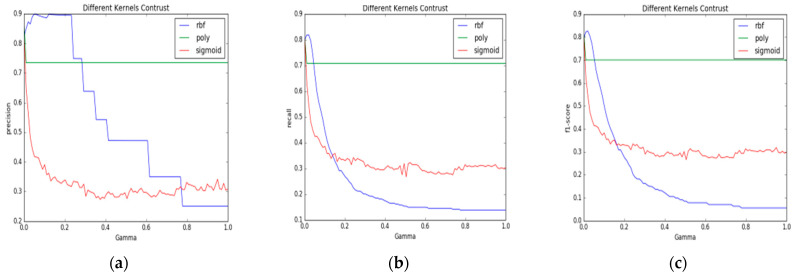
The relationship between SVM model performance and parameters after pre-LDA processing. (**a**) Accuracy; (**b**) recall rate; (**c**) *f*1 value.

The crucial step in establishing the SVM classifier model is identifying the kernel function and its coefficient for the support vector machine. After undergoing LDA processing, the data distribution of the pig face images is expected to change in an identical manner. It implies that proceeding with the tests using the parameters established previously may no longer yield the optimal outcomes. Consequently, it is imperative to redetermine the kernel function and its coefficient for the SVM model. Employing the testing method outlined in “SVM model parameter de-termination”, the kernel function and its coefficient for the SVM model are definitively established, with the input being pig face images that have received LDA treatment. The performance of different kernel functions and coefficients was compared, revealing relationships depicted in Figure 6.

In Figure 6a, the *x*-axis represents the coefficients for three kernel functions: polynomial, radial basis (RBF), and sigmoid, ranging from 0.0 to 1.0. The *y*-axis denotes the precision values, also ranging from 0.0 to 1.0. The figure reveals that the RBF kernel function achieves a peak precision of 90% when its coefficient is small, particularly within the range of 0.05 to 0.25. Beyond this range, the precision declines sharply, dropping below 50% at coefficients around 0.4. Conversely, the polynomial kernel function demonstrates a stable precision of approximately 70% across most coefficient values, stabilizing notably when the coefficient is set at 0.03. The sigmoid kernel function shows a weaker performance, achieving a maximum precision of around 70% when the coefficient is approximately 0.01. As the coefficient increases, the precision of the sigmoid kernel function declines steadily to around 35%. According to the above analysis available, the coefficient should be determined at about 0.05.

Figure 6b,c depict the relationships between kernel function coefficients and the recall and f1-score metrics, respectively. The trends are consistent across all three kernel functions. For the RBF kernel function, the recall reaches its peak at 83% when the coefficient is set to around 0.03. Beyond this value, the recall decreases rapidly, stabilizing at lower values (10% to 20%) for coefficients above 0.5. Similarly, the f1 score for the RBF kernel function aligns closely with its recall, reflecting the trade-off between precision and recall. The polynomial kernel function, in contrast, stabilizes quickly at approximately 70% for both recall and f1-score metrics, indicating a robust performance across a range of coefficients. The sigmoid kernel function shows a marked decline in both recall and f1 score as its coefficient increases, stabilizing at approximately 35% when the coefficient exceeds 0.04. These results suggest that the RBF kernel function, with a coefficient of 0.03, offers the best overall performance in terms of recall and f1 score. Based on the above factors, in this paper, the radial basis function (RBF) is chosen as the kernel function, and the coefficient is set to 0.03 and is used as the test parameter value of the SVM validation set.

#### 3.2.3. Hyperparameter Optimization of the Random Forest Model

In this study, we continued to use the Random Forest (RF) model configuration that was optimized and validated in previous research, “Study on the Influence of PCA Preprocessing on Pig Face Identification with Random Forest.” This decision was based on two studies that used the same dataset but employed different preprocessing techniques (PCA and LDA). In the previous study, we thoroughly tested the effect of varying the number of decision trees on the performance of the RF model and found that with 65 decision trees, the model achieved the highest classification accuracy of 90.61% while maintaining the processing time within an acceptable range.

During the process of hyperparameter optimization for the random forest (RF) model, we systematically tested the impact of varying the number of decision trees from 10 to 100 on the model’s performance. We found that when the number of decision trees increased to 65, the model’s classification accuracy reached its peak. However, further increasing the number of decision trees led to a diminishing return in accuracy improvement while significantly increasing the computation time. This process was validated through multiple experiments, and the relevant results were detailed in charts to ensure the reliability and transparency of the findings. Based on these discoveries, we decided to directly apply these optimized parameter settings to the current study, aiming to ensure consistency in the research methodology and to more accurately assess the effect of LDA preprocessing compared to PCA preprocessing. This approach not only reflects the rigor of scientific research but also aligns with the principles of efficiency and resource optimization.

#### 3.2.4. Model Evaluation Index of Optimization Plan

**Figure 7:**
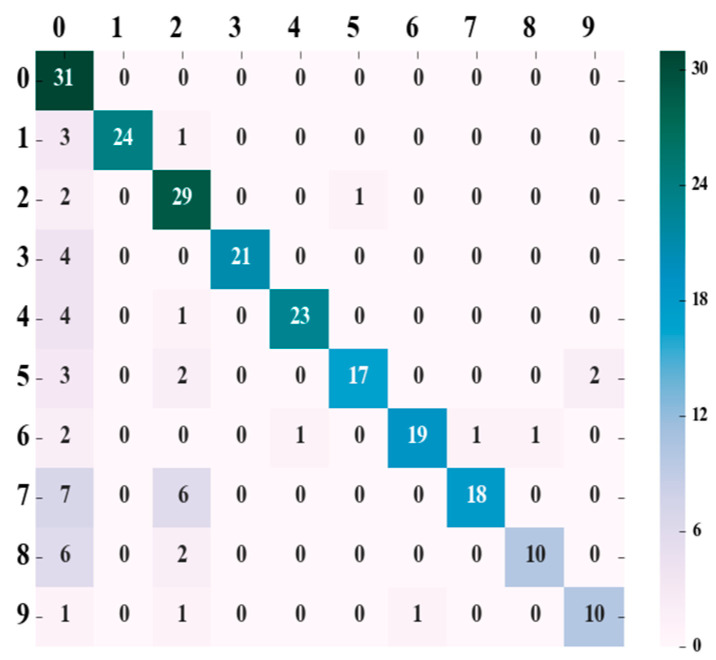
SVM + LDA prediction result confusion matrix.

After determining the parameters in the training set, with the radial basis function (RBF) selected as the kernel function for the SVM, a coefficient of 0.01 was used for the tests conducted on the validation set. The prediction accuracy, recall rate, and *f*1 value of each category were calculated, and a confusion matrix was generated, as shown in Figure 7. In this matrix, the left-most column represents the actual categories; the top row indicates the predicted categories; the main diagonal illustrates the number of correct predictions per category, and the right-most column provides a visual representation of the correct values for each category involved in the predictions.

Figure 7 illustrates the confusion matrix for the SVM model after LDA preprocessing. The rows represent true classes, and the columns represent predicted classes, with diagonal elements indicating correct classifications and off-diagonal elements showing misclassifications. Compared to Figure 5, the classification performance significantly improves, particularly for classes that had higher misclassification rates, such as class 9. For example, the misclassification rate for class 9 has decreased notably, suggesting that LDA has helped the model better differentiate between these classes. Similarly, the misclassification rate for class 5 has been reduced, with fewer instances incorrectly classified as class 4, highlighting the positive impact of LDA in improving the model’s ability to distinguish between challenging categories.

Building on these improvements observed in Figure 7, we calculated the precision, recall, and *f*1-score values for each of the 10 distinct pigs, based on the confusion matrix data from Figure 7, as shown in Table 4. These metrics, derived from Formulas (10)–(12), further demonstrate the enhanced classification performance following LDA preprocessing and offer a more comprehensive evaluation of the model’s ability to handle different classes.

The SVM classifier, enhanced by LDA preprocessing, was employed for the identification of individual pigs. This approach not only heightened the accuracy of identification but also diminished both the training and testing durations of the model. Detailed test indices for both schemes are presented in Table 5.

It can be seen from Table 5 that the accuracy rate of the LDA + SVM test program reached 86.3%, which is 2.64 percentage points higher than that of the single SVM recognition; this occurs because LDA extracts the main features and ignores the secondary features and noise effects, which improves the recognition accuracy to a certain extent. The operating efficiency of the LDA + SVM test algorithm has been greatly improved. Its training time is 98 ms, while the single SVM solution is 12,823 ms, which is 0.7% of the SVM’s time. The recognition time is 1 ms, and the SVM solution recognition time is 329 ms, which is 0.3% of the SVM’s time; the operating efficiency improved significantly.

In this study, we selected traditional machine learning models such as the SVM, RF, KNN, LightGBM, and MLP as candidate base models. These models have shown excellent performance across various tasks, but each model has certain limitations. For example, LightGBM [61,62], while efficient and scalable in handling high-dimensional features and large datasets, is complex, containing many hyperparameters (e.g., tree depth, number of leaf nodes, and learning rate), making the tuning process complex and time-consuming. Additionally, k-nearest neighbors (kNNs) [63,64], although a simple and effective classification method, is susceptible to noise, especially in high-dimensional data, where noise may lead to misclassification. Multilayer perceptron (MLP) [65,66], on the other hand, requires a large amount of data to perform well, has a relatively slow training process, and is prone to overfitting. Moreover, the presence of many hyperparameters such as the number of hidden layers, neurons, and learning rate makes the tuning process complex, significantly impacting the model’s performance. In contrast, random forest (RF) and the support vector machine (SVM) excel in handling noisy data, stability, and generalization capabilities, making them more suitable for the needs of this study.

To investigate the impact of LDA preprocessing on the performance of machine learning models in individual pig recognition, the operational efficiency of models using SVM, RF, KNN, MLP, LDA + SVM, PCA + SVM, LDA + RF, PCA + RF, LDA + KNN, PCA + KNN, LDA + MLP, and PCA + MLP was comparatively analyzed through experiments. The findings are detailed in Table 6.

Table 6 provides a comprehensive comparison of various methods used for pig face recognition, including SVM, RF, KNN, MLP, LDA + SVM, and PCA + SVM. Among these, SVM, LDA + SVM, and PCA + SVM are highlighted in this study as representative methods for detailed analysis due to their balance between simplicity, efficiency, and accuracy. The table demonstrates that PCA + SVM achieves the highest accuracy of 88.85%, making it suitable for scenarios prioritizing precision. However, its computational demands are significantly higher, with a testing time of 69 ms and a training time of 3861 ms. LDA + SVM, in contrast, offers a competitive accuracy of 86.30%, while drastically reducing testing time to 1 ms and training time to 98 ms. These attributes make LDA + SVM particularly suitable for real-time and resource-constrained applications, such as embedded systems. SVM, while the simplest method, serves as a baseline, achieving an accuracy of 83.66%, but it comes with higher computational costs compared to LDA + SVM. The results in Table 6 highlight the trade-offs between computational efficiency and accuracy, offering insights into the optimal application scenarios for each method.

**Figure 8:**
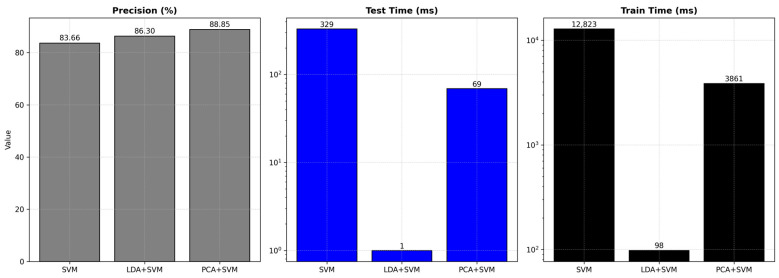
Comparison of generalization test results for the selected methods.

To more intuitively compare the effects of the methods used, a bar chart is introduced, as shown in Figure 8. This figure focuses on the comparative performance of SVM, LDA + SVM, and PCA + SVM in terms of accuracy, testing time, and training time.

Figure 8 highlights the trade-offs between these three methods. PCA + SVM achieves the highest accuracy at 88.85%, surpassing LDA + SVM by 2.55 percentage points and SVM by 5.19 percentage points. However, this accuracy comes at the cost of significantly increased testing and training times (69 ms and 3861 ms, respectively). In contrast, LDA + SVM provides a competitive accuracy of 86.30% while maintaining exceptional efficiency, with testing time reduced to just 1 ms and training time to 98 ms. These findings underscore LDA + SVM’s suitability for scenarios requiring both accuracy and efficiency, particularly in real-time pig face recognition systems. On the other hand, PCA + SVM is more appropriate for resource-rich environments where higher accuracy is paramount.

Furthermore, in additional research by [42,43,44,67,68,69,70,71,72], the investigators examined the efficacy of cutting-edge neural networks, such as AlexNet, YOLO, and tiny-YOLO, in the contexts of porcine identification, craniofacial posturing, and ethological assessments. Nevertheless, the extensive parameterization and deep-layered architecture of modern neural networks pose significant constraints, hindering their application within embedded systems. This challenge serves as a primary impetus for the current study.

## 4. Discussion

This study mainly studies the effect of linear discriminant analysis (LDA) preprocessing on the efficiency of support vector machine (SVM) pig face recognition. The effectiveness, limitations, and particularity of the experimental process of this study are discussed as follows:

(1) Discussion on the Effectiveness of the Experimental Results:

In theory, linear discriminant analysis (LDA) effectively improves the performance of classification tasks via dimensionality reduction and denoising [73]. This method brings superior speed and accuracy performance, which is also verified by the experimental results. The training time and test time are reduced to 0.7% and 0.3% of the original, respectively. The research shows that [74] although this preprocessing method will increase the computational overhead, it will improve the classification accuracy. In this study, the classification accuracy is increased by 2.64%. In addition, in many practical applications, the efficiency of LDA is much higher than its computational cost [75], which is also confirmed by the results of this study.

At present, pig individual recognition technology is developing in the direction of deep learning. Wang et al. [56] developed a cascade neural network model with high accuracy, which is suitable for large and medium-sized pig farms. Mathieu Marsot et al. [55] used a classifier based on Haar features and a shallow convolutional neural network to achieve an accuracy of 86.3% in pig individual recognition. Wang Rong et al. [76] proposed PigFaceNet, which has a recognition accuracy of 94.28%. These research results can be applied to large and medium-sized farms. Under this technical background, this study is suitable for the intelligent management of small and medium-sized pig farms because it greatly improves the time efficiency and reduces the complexity of the calculation model.

(2) Discussion on the Limitations of the Experimental Results:

The small sample test data may have certain limitations on the universality of the test results, and the results may be different during multiple tests. However, studies have shown that [77] expanding the sample size through data augmentation technology can effectively reduce the impact of small samples and improve the robustness of the model to the environment, which is also the main reason for the use of data augmentation in this study. In addition, small sample datasets also have their specific applicable scenarios. Chen et al. [78] proposed an LDA-based face recognition system based on small datasets and achieved good application results. Jingjing Cao et al. [79] collected 480 samples for eight species in the study of mangrove species identification, and the classification accuracy was 96.5%, which showed good recognition effect in its application. The results of this study have certain application value in the intelligent management of small and medium-sized pig farms.

(3) Discussion on the Specificities of the Experimental Process:

In the experiment of directly using SVM for classification, we observed that the classification accuracy, recall rate and *f*1 value of the three kernel functions remained stable with the change in Gamma parameter, showing a straight line. This phenomenon may be closely related to the nature of data characteristics. Specifically, the original features in the pig face image data have relatively limited discrimination between categories, and there may be a certain degree of noise and redundant features in the data. These factors may weaken the influence of Gamma parameters on the model performance, resulting in a stable curve change. It should be pointed out that this is not the defect of the data but the embodiment of its natural attributes, reflecting the complexity and diversity of the data itself. However, after introducing LDA for dimensionality reduction, the discrimination of pig face features was enhanced. When SVM was used again for classification, we noticed that the classification curve changed significantly within the same Gamma parameter range. This phenomenon shows that LDA effectively optimizes the data features so that the kernel function can more fully capture the relationship between categories, thereby improving the sensitivity of SVM to Gamma parameter changes.

## 5. Conclusions

This paper has explored the efficacy of linear discriminant analysis (LDA) as a preprocessing step to enhance the performance of pig face identification using support vector machines (SVMs). Through rigorous experimentation, we identified the optimal kernel function and its coefficient for the SVM classifier. A comparative analysis of the identification performance between two methodologies, one employing SVM alone and the other integrating LDA with SVM, presents the following conclusions:

(1) For pig individual recognition, different preprocessing methods have different degrees of positive effects on different traditional machine learning methods. LDA has the greatest impact on SVM and the most obvious improvement in time efficiency;
(2) In the pig face recognition task, the choice of kernel function has a significant impact on the classification results. The experimental results show that when combined with the LDA preprocessing method and SVM classifier, the recognition effect of RBF kernel function is better than that of polynomial kernel (Poly) and sigmoid kernel;
(3) By increasing the LDA preprocessing method, the SVM is used to improve the operation efficiency of pig individual identification, making it more suitable for the development and application of mobile terminals and embedded devices and more suitable for the management of small and medium-sized pig farms.

## Figures and Tables

**Table 1:** Named terms list.

Abbreviation	Meaning
LDA	Linear Discriminant Analysis
LLE	Local Linear Embedding
RBF	Radial Basis Function
SVM	Support Vector Machine
YOLO	You Only Look Once
RF	Random Forest
Faster-rcnn	Faster Region-based Convolutional Neural Networks
KNN	K-Nearest Neighbors
SSD	Single Shot Detector
PCA	Principal Component Analysis
UHFRFID	Ultra-High Frequency Radio Frequency IDentification
RFID	Radio Frequency IDentification
HFRFID	High-Frequency Radio Frequency IDentification
ICA	Independent Component Analysis
t-SNE	t-Distributed Stochastic Neighbor Embedding
MDS	Multidimensional Scaling
MLP	Multilayer Perceptron
LightGBM	Light Gradient Boosting Machine

**Table 2:** Model training parameters.

Model Parameter	Numerical Value
kernel function	RBF
C	1.0
degree	3
tol	1 × 10^−3^
cache_size	200
max_iter	100

**Table 3:** Prediction performance table for the SVM model.

Category	Precision (%)	Recall (%)	f1-Score (%)	Count [a]
1	88	90	89	31
2	96	82	88	28
3	95	62	75	32
4	82	92	87	25
5	76	79	77	28
6	82	58	68	24
7	62	88	72	24
8	96	71	81	31
9	89	89	89	18
10	46	100	63	13
average	83.66	79.53	79.95	25

**Table 4:** SVM + LDA model prediction performance table.

Category	Precision (%)	Recall (%)	*f*1-Score (%)	Count [a]
1	49	100	66	31
2	100	86	92	28
3	69	91	78	32
4	100	84	91	25
5	96	82	88	28
6	94	71	81	24
7	95	79	86	24
8	95	58	72	31
9	91	56	69	18
10	83	77	80	13
average	86.30	79.53	80.42	25

**Table 5:** The optimization result of the SVM model via LDA preprocessing.

Model	Precision (%)	Precision Change	Test_Time (ms)	Test_New/Old (%)	Train_Time (ms)	Traintest_New/Old (%)
SVM	83.66	0	329	100	12,823	100
LDA + SVM	86.30	+2.64	1	0.30	98	0.70

**Table 6:** Comparison of generalization test results.

Model	Precision (%)	Precision Change	Test_Time (ms)	Test_New/Old (%)	Train_Time (ms)	Traintest_New/Old (%)
SVM	83.66	0	329	100	12,823	100
RF	90.61	0	8	100	1229	100
KNN	89.76	0	19	100	651	100
MLP	87.89	0	196	100	1523	100
LDA + SVM	86.30	+2.64	1	0.3	98	0.7
PCA + SVM	88.85	+5.19	69	20.9	3861	30.1
LDA + RF	85.17	−5.44	6	75	100	8.1
PCA + RF	93.22	+2.61	6	75	1340	109
LDA + KNN	87.91	−1.85	13	68	175	27
PCA + KNN	89.98	+0.22	10	53	156	24
LDA + KNN	88.32	+0.43	153	78	990	65
PCA + KNN	89.12	+1.23	132	67	868	57

## Data Availability

Our research team is very grateful for the dedicated support provided by Professor Yan Hongwen in data collection, as well as the assistance in experimental design throughout the entire research process.
